# Evaluating the outcomes of a podiatry-led assessment service in a public hospital orthopaedic unit

**DOI:** 10.1186/s13047-014-0045-6

**Published:** 2014-11-18

**Authors:** Daniel R Bonanno, Virginia G Medica, Daphne S Tan, Anita A Spring, Adam R Bird, Jana Gazarek

**Affiliations:** Department of Podiatry, Faculty of Health Sciences, La Trobe University, Bundoora, VIC 3086 Australia; Lower Extremity and Gait Studies program, Faculty of Health Sciences, La Trobe University, Bundoora, VIC 3086 Australia; Podiatry Department, The Northern Hospital, Epping, VIC 3076 Australia

## Abstract

**Background:**

In Australia, the demand for foot and ankle orthopaedic services in public health settings currently outweighs capacity. Introducing experienced allied health professionals into orthopaedic units to initiate the triage, assessment and management of patients has been proposed to help meet demand. The aim of this study was to evaluate the effect of introducing a podiatry-led assessment service in a public hospital orthopaedic unit. The outcomes of interest were determining: the proportion of patients discharged without requiring an orthopaedic appointment, agreement in diagnosis between the patient referral and the assessing podiatrist, the proportion of foot and ankle conditions presenting to the service, and the proportion of each condition to require an orthopaedic appointment.

**Methods:**

This study audited the first 100 patients to receive an appointment at a new podiatry-led assessment service. The podiatrist triaged ‘Category 3’ referrals consisting of musculoskeletal foot and ankle conditions and appointments were provided for those considered likely to benefit from non-surgical management. Following assessment, patients were referred to an appropriate healthcare professional or were discharged. At the initial appointment or following a period of care, patients were discharged if non-surgical management was successful, surgery was not indicated, patients did not want surgery, and if patient’s failed to attend their appointments. All other patients were referred for an orthopaedic consultation as indicated.

**Results:**

Ninety-five of the 100 patients (69 females and 31 males; mean age 51.9, SD 16.4 years) attended their appointment at the podiatry-led assessment service. The 95 referrals contained a total of 107 diagnoses, of which the podiatrist agreed with the diagnosis stated on the referral in 56 cases (Kappa =0.49, SE = 0.05). Overall, 34 of the 100 patients were referred to an orthopaedic surgeon and the remaining 66 patients were discharged from the orthopaedic waiting list without requiring an orthopaedic consultation.

**Conclusions:**

Two-thirds of patients who had an appointment at the podiatry-led assessment service were discharged without requiring a surgical consultation. The introduction of a podiatry-led service assists with timely provision of patient care and ensures those with the greatest need for orthopaedic surgery have improved access to specialist care.

## Background

Foot pain affects approximately one in five people in the general community and it is associated with reduced health-related quality of life [1] and self-reported disability [2]. Although many musculoskeletal foot conditions can be managed with non-surgical interventions [3,4], recalcitrant and complex cases can require surgery [5]. In Australia, the demand for foot and ankle orthopaedic services in public health settings currently outweighs capacity due to factors such as limited theatre availability, competing surgical priorities and a limited surgical workforce [6]. With a growing and ageing population in Australia, the future demand for foot and ankle surgery is expected to increase [7].

Several initiatives have been proposed to help overcome the problem of unmet surgical demand in orthopaedic units [6], with one being the introduction of experienced allied health professionals to perform some roles traditionally provided by orthopaedic surgeons [8–11]. Allied health professionals in these positions require specialty area expertise, often have completed relevant post-graduate studies, and have the competency to maximise their scope of practice in a public outpatient setting [8–11]. The roles they perform can include triaging orthopaedic referrals, assessing patients, and establishing and initiating a management plan [8–11]. Introducing experienced allied health professionals into orthopaedic units can be relatively seamless to implement as it utilises the skills of an existing workforce [6]. The current evidence for using allied health professionals in these roles is generally promising with studies indicating high patient and referrer satisfaction [9,11–14], reductions in waiting time [8,11], high agreement in diagnosis and management decisions with orthopaedic surgeons [9,15], and a high proportion of patients being assessed, managed and discharged without requiring a surgical consultation [8,9,11].

Although the use of allied health professionals in orthopaedic clinics is well documented, only two studies have investigated the role of a podiatrist working in this capacity in Australia; and they have demonstrated reductions in patient waiting times [8], improved service efficiency and high patient satisfaction [14]. The aim of this clinical audit was to evaluate the outcomes of introducing a podiatrist to triage, assess and initiate the management of patients referred to an orthopaedic outpatient unit with a foot or ankle condition. The primary outcome was to determine the proportion of patients that had an appointment at the podiatry-led assessment service to be discharged without requiring an orthopaedic appointment. Secondary outcomes include determining the: (i) agreement in diagnosis written on the patient referral compared with that of the assessing podiatrist, (ii) proportion of foot and ankle conditions presenting to the service, and (iii) proportion of each condition to require an orthopaedic appointment.

## Methods

This study was a retrospective clinical audit of the first 100 patients to receive an appointment at a new podiatry-led assessment service in the orthopaedic outpatient department at The Northern Hospital, a public hospital located in an outer northern suburb of Melbourne. The podiatrist (DRB) responsible for triaging referrals and assessing patients has over 10 years of clinical experience, completed postgraduate studies (Postgraduate Diploma in Research Methodology) and has several peer-reviewed publications relating to the assessment and management of musculoskeletal conditions of the foot and lower limb.

Patients referred for a surgical consultation were initially triaged by orthopaedic surgeons into different categories of surgical priority (Table 1). Referrals were classified as ‘Category 3’ if the condition was considered unlikely to deteriorate quickly or had no potential to become an emergency. The podiatrist subsequently triaged all existing and new ‘Category 3’ referrals consisting of musculoskeletal foot and ankle conditions. Those considered likely to benefit from non-surgical management were provided with an appointment at the podiatry-led assessment service.

**Table 1:** **Hospital waiting list categories**

**Category**	**Description**	**Recommended admission time**
1	Patient has a condition that has potential to deteriorate rapidly to the point it may become an emergency	Within 30 days
2	Patient has a condition that is causing some pain, dysfunction or disability, but is unlikely to deteriorate quickly or become an emergency	Within 90 days
3	Patient has a condition that is causing minimal or no pain, dysfunction or disability, which is also unlikely to deteriorate quickly or has no potential to become an emergency	Within 365 days

Patients were independently assessed by the podiatrist, with an orthopaedic surgeon generally being available for consultation if required. A standardised assessment form was used to record health-related information and details specific to the patient’s condition(s) as per standard practice. Patients considered likely to benefit from non-surgical interventions were referred to an appropriate healthcare professional, as determined by the assessing podiatrist, and a management plan was established. Due to the variety of conditions expected to be seen, no standardised treatment protocols were utilised and all initial management decisions were based on the podiatrist’s clinical reasoning in context of each patient and evidence based practice. Patients identified as requiring a surgical opinion were escalated for an appointment with an orthopaedic surgeon.

Patients were discharged from the orthopaedic waitlist with patient consent when: (i) non-surgical treatment was successful in resolving the presenting problem, (ii) if surgery was not indicated, either for the condition or due to co-morbidities, and (iii) if patients indicated they did not want surgery. In addition, patients were discharged if they failed to attend their appointment on two or more occasions without prior communication. Patients that did not adequately respond to non-surgical interventions were referred for an orthopaedic consultation if clinically indicated.

### Data collection and analysis

All data were obtained from the Northern Health electronic patient records and consisted of information recorded as per standard practice. Data collected included patient demographics, referrer and podiatrist diagnosis, and the patient’s referral and treatment pathway. Inter-rater agreement between the diagnosis provided on the referral and the triaging podiatrist was determined using the Cohen’s kappa statistic. All data were de-identified prior to analysis and were analysed using IBM SPSS (version 21.0). This clinical audit was considered a quality assurance activity that did not require ethical review in accordance with the National Statement on Conduct in Human Research (NHMRC 2007).

## Results

### Profile of patients and conditions

**Figure 1:**
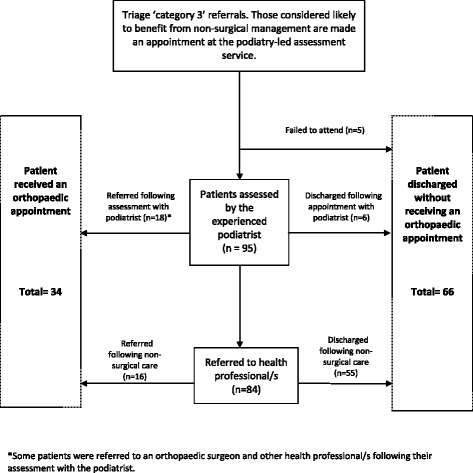
**Flowchart of patients attending the podiatry-led assessment service.**

The 100 patients were provided an appointment at the podiatry-led assessment service between June 2012 and April 2013. The patients consisted of 69 females and 31 males, with a mean age of 51.9 (SD 16.4) years. Of the 100 patients, five failed to attend their appointment on two or more occasions and were discharged (Figure 1). Of the remaining 95 patients, their referrals contained a total of 107 diagnoses (some patients had more than one diagnosis), of which 56 were agreed upon by the triaging podiatrist (Kappa = 0.49, SE = 0.05). The podiatrist diagnosed 20 different conditions, with the most common diagnoses being hallux valgus (n = 23), plantar fasciitis (n = 21), inter-metatarsal neuroma or bursitis (n = 13), tibialis posterior tendinopathy (n = 7), and Achilles tendinopathy (n = 5). A summary of all diagnoses (n = 104) made by the podiatrist is provided in Table 2.

**Table 2:** **Diagnoses (n = 104) of patients (n = 95) as determined by the podiatrist and the number of each diagnosis to require an orthopaedic appointment**

**Condition**	**Number of presentations n (% of total diagnoses)**	**Required orthopaedic appointment n (% per condition)**	**Did not require orthopaedic appointment n (% per condition)**
Hallux valgus	23 (22.1%)	9 (39.1%)	14 (60.9%)
Plantar fasciitis	21 (20.1%)	3 (14.3%)	18 (85.7%)
Inter-metatarsal neuroma/bursitis	13 (12.5%)	5 (38.5%)	8 (61.5%)
Tibialis posterior tendinopathy	7 (6.7%)	1 (14.3%)	6 (85.7%)
Achilles tendinopathy	5 (4.8%)	1 (20.0%)	4 (80%)
Plantar plate tear	4 (3.8%)	2 (50.0%)	2 (50%)
Neuralgic pain (origin extrinsic to foot)	4 (3.8%)	1 (25.0%)	3 (75%)
Hallux rigidus	3 (2.9%)	3 (100%)	0 (0%)
Midfoot osteoarthritis	3 (2.9%)	1 (33.3%)	2 (66.6%)
Digital deformity	3 (2.9%)	1 (33.3%)	2 (66.6%)
Degenerative arthropathy	3 (2.9%)	3 (100%)	0 (0%)
Peroneal tendinopathy	3 (2.9%)	1 (33.3%)	2 (66.6%)
Synovitis/capsulitis	2 (1.9%)	1 (50.0%)	1 (50%)
Inflammatory arthritis	2 (1.9%)	1 (50.0%)	1 (50%)
Dermatological	2 (1.9%)	0 (0%)	2 (100%)
Sesamoiditis	1 (1.0%)	0 (0%)	1 (100%)
Ganglion	1 (1.0%)	1 (100.0%)	0 (0%)
Retro-calcaneal bursitis	1 (1.0%)	1 (100.0%)	0 (0%)
Sub-ungual exostosis	1 (1.0%)	1 (100.0%)	0 (0%)
Extensor tendinopathy	1 (1.0%)	0 (0%)	1 (100%)
Diagnosis not determined	1 (1.0%)	0 (0%)	1 (100%)
**Total**	**104 (100%)**	**36 (34.6%)**	**68 (65.4%)**

### Patient destination

Following the assessment of the 95 patients at the podiatry-led assessment service, 6 were discharged without receiving further treatment or requiring further referral(s). Of the remaining 89 patients, a total of 102 referrals were made following the initial appointment. Referrals were sent to a podiatrist (n = 72), orthopaedic surgeon (n = 18), orthotist (n = 5), physiotherapist (n = 3), diabetes educator (n = 1), occupational therapist (n = 1), gerontologist (n = 1) and rheumatologist (n = 1). After a period of receiving non-surgical care, a further 16 patients were referred to an orthopaedic surgeon. Overall, a total of 34 of the 100 patients were referred to an orthopaedic surgeon and the remaining 66 patients were discharged from the orthopaedic waiting list without requiring an orthopaedic consultation (Figure 1).

### Surgical referral for various conditions

The 34 patients referred for a surgical opinion were diagnosed by the podiatrist with 17 different conditions. Of interest, 15 of these patients had a diagnosis stated on their initial referral that was not agreed upon by the podiatrist. The most common diagnoses provided by the podiatrist that was referred for a surgical opinion were hallux valgus (n = 9), inter-metatarsal neuroma or bursitis (n = 5), plantar fasciitis (n = 3), hallux rigidus (n = 3), a variety of arthropathies (n = 3) and plantar plate tears (n = 2) (Table 2).

## Discussion

This clinical audit evaluated the effects of introducing an experienced podiatrist to initiate the triage, assessment and management of patients referred to an orthopaedic outpatient unit with a foot or ankle condition. The findings of this study indicate that two-thirds of the patients who had an appointment at the podiatry-led assessment service were removed from the orthopaedic waitlist without requiring a surgical consultation. These findings are similar, albeit greater, to previous studies involving podiatrists performing assessment and triage roles where 41-45% of patients were discharged without requiring surgical management [8,14]. Current literature suggests allied health professionals, including podiatrists, can ease demands placed on orthopaedic outpatient services [8,9,14,16]. Importantly, as ‘Category 3’ patients generally wait the longest for a surgical consultation, the assessment and triage service allows those considered likely to benefit from non-surgical interventions to be redirected, in many cases earlier, for effective treatment.

The triage of referrals is intended to ensure patients receive appropriate and timely care, yet this process is highly reliant on referrals being accurate and informative. The results from this study indicate that almost half of the referrals received provided a non-specific diagnosis or one that wasn’t agreed upon by the triaging podiatrist. Although many of the diagnostic disagreements between the referrer and the assessing podiatrist are likely to be true differences in opinion, there is the possibility that in some cases the condition may have changed in the time between the date of the referral and initial appointment. When disagreement is present, it often remains uncertain which diagnosis is most accurate as a definitive diagnosis is not always established. With this in mind, it should be noted that the triaging podiatrist was more likely to provide a more specific diagnosis than that contained on the referrals. Although all patients provided an appointment at the podiatry-led assessment service were considered likely to benefit from non-surgical intervention based on their referral, approximately 20% were directly referred to a surgeon following their initial assessment. In addition, nearly half of all patient referrals to receive an appointment with a surgeon were found to have a non-specific diagnosis or one that wasn’t agreed upon by the podiatrist. This demonstrates the propensity for a referral lacking detail and/or accuracy to delay appropriate surgical treatment when indicated. Based on our findings, we recommend referrers ensure they provide informative and specific referrals to maximise triage efficiency and accuracy.

A novel aspect of the present study is it reported the specific conditions seen at the podiatry-led assessment service and the proportion of these conditions that were discharged with and without requiring a surgical opinion. This information can potentially be used in several ways. First, referrers could be advised that specific foot conditions, such as plantar fasciitis, should be referred for non-surgical management as a first-line of care as supported by this study and, more importantly, high quality clinical trials and clinical practice guidelines [4,17,18]. This would allow patients to receive appropriate, timely care and reduce unnecessary referrals to orthopaedic services, with the latter further reducing demand on surgical services. In contrast, for conditions such as hallux rigidus, it would be reasonable for referrers to strongly consider a surgical referral if the condition remains recalcitrant following non-surgical management [5,19]. Second, when all new referrals are being triaged, conditions likely to benefit from non-surgical and surgical interventions could be identified and the most appropriate treatment options and referrals could be established. Importantly, this information could be applied at various stages of triage including the initial entry points into a public health service. If these changes were implemented it could potentially improve the efficiency of how foot and ankle referrals are triaged and managed.

Although the preliminary findings of this audit are promising, further service efficiencies are likely and should be considered. As triage prioritisation systems have been shown to have issues with reliability [20,21], podiatrists could potentially triage all referrals with foot and ankle conditions to assist with identifying prioritisation categories and provide patients with early intervention where possible. By reducing the demands of triage on orthopaedic surgeons, it could potentially allow surgeons more time to consult patients already triaged and identified as requiring their surgical skills.

The findings of this audit should be considered in light of several limitations. First, and most importantly, the lack of a comparison group introduces uncertainty around whether the results reported are the effect of the new podiatry-led assessment service or unrelated factors. In addition, as assessment and patient care was not controlled, the results of this audit may be influenced by the individual clinicians rather than the new initiative itself. Nevertheless, in light of the limitations, the findings of this audit can be viewed as additional evidence to support the introduction of podiatry-led assessment services in orthopaedic units. In addition to addressing the aforementioned limitations, future research should consider measuring outcomes such as cost-benefit, waiting times, patient and referrer satisfaction and validated measures of changes in pain, function and psychological well-being.

## Conclusion

The majority of patients referred to an orthopaedic outpatient unit with a foot or ankle condition who were provided an appointment at the podiatry-led assessment service were discharged without requiring a surgical appointment. The introduction of the podiatry-led assessment service may assist with improving the efficiency of care, which is provided by the most appropriate health professional, to patients referred for a surgical consultation with a foot and ankle condition.
